# Effect of Incorporating Titanium Dioxide Nanoparticles into White Portland Cement, White Mineral Trioxide Aggregate, and Calcium Enriched Mixture Cement on the Push-out Bond Strength to Furcal Area Dentin

**DOI:** 10.30476/dentjods.2023.92290.1627

**Published:** 2023-12-01

**Authors:** Shahriar Shahi, Mohammad Samiei, Mahmoud Bahari, Hamidreza Yavari, Mona Rahbar Mahvarian

**Affiliations:** 1 Dental and Periodontal Research Center, Tabriz University of Medical Sciences, Tabriz, Iran; 2 Dept. of Endodontics, Faculty of Dentistry, Tabriz University of Medical Sciences, Tabriz, Iran

**Keywords:** Calcium-enriched mixture cement, Mineral trioxide aggregate, Portland cement, Titanium dioxide

## Abstract

**Statement of the Problem:**

Bond strength of furcation repair materials is an essential factor in clinical success. Studies on the effect of adding titanium dioxide (TiO_2_) nanoparticles on the push-out bond strength of commonly used endodontic cements for furcation perforation repair is limited.

**Purpose:**

This study aimed to evaluate the effect of adding TiO_2_ nanoparticles to white Portland cement (PC), white mineral trioxide aggregate (MTA), and calcium enriched mixture cement (CEM) on their push-out bond strengths.

**Materials and Method:**

In this *in vitro* study, 120 endodontically treated molars were assigned to six groups (n=20) based on the material used to repair the perforation. In three groups, the cements (white PC, white MTA, and CEM) were placed in pure form, and in the three remaining groups, 1 weight % of TiO_2_ was added. The push-out bond strength was measured using a universal testing machine at a strain rate of 0.5 mm/min. Data were analyzed using one-way ANOVA and post hoc Games-Howell test (*p*< 0.05).

**Results:**

One-way ANOVA showed significant differences in the mean bond strength values between the six groups (*p*= 0.002). The post hoc Games-Howell test showed that the bond strengths in MTA+TiO_2_ and PC+TiO_2_ groups were significantly higher than those in MTA and PC groups, respectively. However, there was no significant difference in the bond strength between CEM and CEM+ TiO_2_ groups.

**Conclusion:**

The incorporation of TiO_2_ into MTA and PC increased their push-out bond strength. However, it did not affect the push-out bond strength of CEM cement.

## Introduction

Mineral trioxide aggregate (MTA), Portland cement (PC), and calcium enriched mixture (CEM) cement are widely used as biomaterials in the perforation repair, root-canal obturation, pulpotomy, and apexification procedures [ 1 - 2 ]. MTA, as a bioactive material, forms a hydroxyapatite or carbonate apatite layer on its surface in the presence of tissue fluid, due to hydration. This interfacial layer creates a chemical bond between MTA and the furcation wall [ 3 - 4 ], which improves the sealing ability and marginal adaptation of MTA [ 5 ]. The main composition of PC and MTA is similar [ 6 ]. PC’s ability to seal furcal perforations is similar to or even more effective than MTA [ 7 ]. PC has been suggested as a proper substitute for MTA due to its lower price, better sealing ability, and biocompatibility [ 8 ].

CEM has recently been introduced and consists of a variety of calcium compounds and is chemically different from MTA and PC [ 9 ]. Its surface is capable of producing hydroxyapatite with exogenous and endogenous sources. It is similar to MTA in expansion during the setting reaction and has been reported to be better than MTA regarding film thickness, flow, and sealing capability [ 9 - 10 ].

Various ingredients including calcium chloride [ 11 ], zeolite-silver-zinc compound [ 12 ], propylene glycol [ 12 ], disodium hydrogen phosphate [ 13 ], chlorhexidine [ 14 ], and titanium dioxide (TiO_2_) [ 15 ] have been added to MTA in order to improve its clinical properties, reduce the setting time, and increase its compressive and pushout bond strengths. They have been successful in enhancing some of its properties and unsuccessful in some others. For example, adding 5% calcium chloride and K-Y gel have reduced the setting time [ 11 ]; adding 0.12% chlorhexidine have increased compressive strength; and the combination of zeolite-silver-zinc have reduced its compressive strength [ 16 ].

TiO_2_ is a metal oxide widely used in everyday life, including its use in wastewater treatment, accelerating chemical reactions, anti-fog layers, self-cleaning glass, and cosmetic products. The superb photoelectric photocatalytic and hydrophilic properties of TiO_2_ nanoparticles as a unique property and its mechanical properties, such as density, melting point, and high elasticity coefficient, result in their consideration as a suitable additive for drugs and biomaterials to increase their efficiency [ 17 ]. Incorporating these nanoparticles into glass-ionomers [ 18 ] and acrylic resins [ 19 ] has improved their mechanical properties in a dose-dependent manner. Mouthwashes containing this compound have antibacterial effects against *streptococcus mutans* and *streptococcus sanguis* [ 20 ].

Samiei *et al*. [ 15 , 21 ] showed that adding 1 w% of TiO_2_ did not adversely affect MTA’s biocompatibility and improved its compressive and push-out bond strengths; however, it increased the working and setting time. Since studies on the effect of adding TiO_2_ nanoparticles to three commonly used endodontic cements on their push-out bond strength is limited, this study aimed to evaluate the impact of incorporating 1 w% of TiO_2_ nanoparticles to white PC, white MTA, and CEM cement on their push-out bond strength in furcation area dentin.

## Materials and Method

In this *in vitro* study, 120 mandibular first molars were selected from the archives of extracted teeth in Department of Oral and Maxillofacial Surgery that had been extracted for periodontal reasons. The protocol was approved by the Ethics Committee of Tabriz University of Medical Sciences (NO: IR.TBZMED.REC.1395.627). The inclusion criteria consisted of absence of cracks, fractures, and caries in the furcation and cervical areas, absence of anomalies in the shape and size of the teeth, and complete formation of tooth roots.

The soft tissues were removed using a hand scaler, and the samples were stored in a physiological saline solution for a maximum of three months until the initiation of the study. The samples were autoclaved immediately before the study began.

The tooth crowns were removed with a diamond disc (Teezkavan, Tehran, Iran) from the CEJ. The teeth were fixed in acrylic resin molds (Acropars, Tehran, Iran) with the furcation area and 3 mm apical to the furcation area exposed to create a space under the furcation to place a matrix to pack cements to repair perforations.

Perforations were created using a $\frac{1}{2}$ round diamond bur (Teezkavan, Tehran, Iran) perpendicular to the furcation area floor and parallel to the tooth long axis. The perforation site was then enlarged using a #4 Peesoreamer (Dentsply Maillefer, Ballaigues, Switzerland) so that the perforation measured 3 mm in diameter. The height of the perforation walls was measured using a periodontal probe. The excess thickness was removed with a diamond disk to leave a height of 2 mm in that area in all the samples. All the samples were irrigated using normal saline solution to eliminate the debris remaining from the working and cutting steps. The samples were divided into six groups (n=20) based on the material used to repair each perforation.

In the group 1, MTA (Angelus Dental Industry Products, Londrina, Brazil) was prepared based on manufacturer’s instructions and located in the perforation area by employing a special carrier. After eliminating excess MTA from the pulp, a wet cotton swab dipped in normal saline solution was placed on the furcation repair material. In the group 2, MTA was mixed with 1 w% of TiO_2_ and placed in the perforation area similar to that in the group 1.

In the group 3, according to the manufacturer’s instructions, a 1:1 ratio of the powder and liquid of CEM cement (BioniqueDent, Tehran, Iran) was mixed and placed in the perforation area; then, wet cotton pellet was placed on the furcation area. In the group 4, 1 w% of TiO_2_ nanoparticle powder was added to CEM cement powder. The rest of the steps were similar to phases performed in the group 3. In the groups 5 and 6, the furcation area perforations were repaired with PC and PC mixed with 1 w% of TiO_2_, respectively. In all the samples, a piece of wet cotton was placed on the cement in the furcation area. Then, the samples were incubated at 37ºC for 72 hours for the complete setting of the cements.

**Figure 1:**
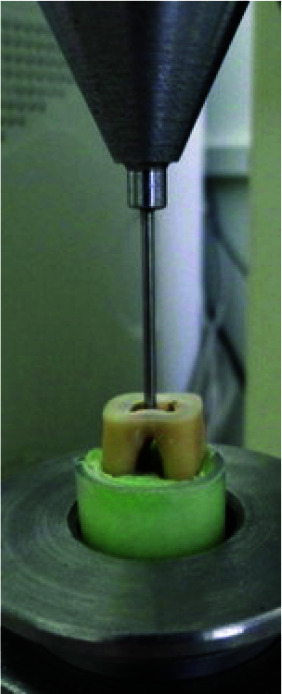
A specimen under push-out test using universal testing machine

Subsequently, the samples were fixed in a steel holder, which was screwed by an aligning device and fixed to its special place on the universal testing machine (Hounsfield Test Equipment, Model H5KS, Surrey, UK). A vertical force was applied using a metal rod 2 mm in diameter at a crosshead speed of 0.5 mm/min directly in the middle of the furcation area cements (Figure1). The maximum force applied to the cement was recorded in Newton before displacement. The push-out bond strength was calculated in MPa by dividing force (N) by the surface area (mm^2^). The following equation was used to calculate the bonded surface area: A= 2πr×h, in which r is the radius of the perforation area and h is the height of the cement in the furcation area in millimeters. After carrying out the push-out bond strength test, the samples were divided into two sections longitudinally using high-speed diamond discs under continuous water and air spray. The failure modes were evaluated under a Nikon stereomicroscope (SMZ 1000, Tokyo, Japan) at 40× magnification [ 15 - 16 ].

One-way ANOVA was used to compare the bond strengths. Post hoc Games-Howell tests were used for two-by-two comparisons of the groups. Statistical significance was set at *p*< 0.05.

## Results

**Figure 2:**
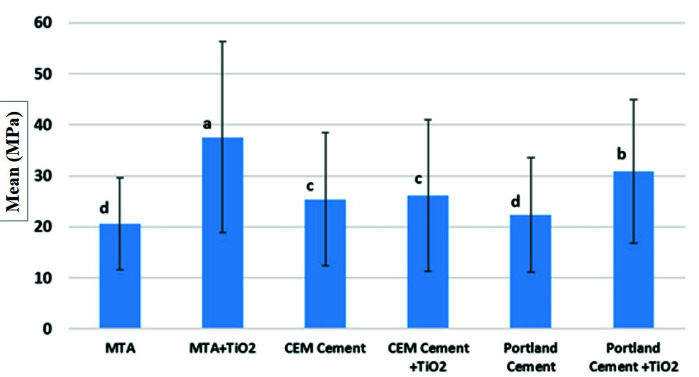
Bar chart showing comparison among study groups. Different letters means statistically significant differences (*p*< 0.05) MTA: Mineral trioxide aggregate, TiO_2_: Titanium dioxide, CEM: Calcium Enriched Mixture

The mean and standard deviation of push-out bond strength values are presented in Table 1. The Shapiro-Wilk test showed that the data were distributed normally in all the groups (*p*> 0.05). One-way ANOVA was used to compare the bond strengths, which showed significant differences in the mean bond strength values between the six groups (*p*= 0.002). Post hoc Games-Howell test was used for two-by-two comparisons of the groups due to non-homogeneity of variances (*p*= 0.03). The results showed that the bond strengths in groups 2 and 6 were significantly higher than that in the groups 1 and 5. However, there was no significant difference in bond strength between the groups 3 and 4. The MTA+TiO_2_ combination exhibited the highest bond strength. The PC+TiO_2_ combination ranked second among the six groups. The CEM cement and CEM cement+TiO_2_ combination ranked third in terms of bond strength. The MTA and PC alone exhibited the lowest bond strength (Figure 2).

**Table 1:** Mean and Standard Deviation (SD) of Push-out bond strength values in study groups

Groups	Push-out Bond Strength (MPa)	
	Mean	SD
MTA	20.60^a^	9.01
MTA + TiO_2_	37.60^b^	18.71
CEM	20.30^a^	13.03
CEM + TiO_2_	26.10^a^	14.82
PC	22.20^a^	11.23
PC + TiO_2_	30.80^b^	14.11

Different superscripts mean statistically significant differences (*p*< 0.05)

MTA: Mineral trioxide aggregate

TiO_2_: Titanium dioxide

CEM: Calcium Enriched Mixture

PC: Portland cement

Evaluation of failure patterns before and after adding TiO_2_ to MTA and PC revealed changes in the failure pattern percentage in the form of a decrease in adhesive failure percentage and an increase in cohesive and mixed pattern percentages. In the case of CEM cement, similar to push-out results, there was no significant change in failure patterns before and after incorporating TiO_2_.

## Discussion

Iatrogenic perforation is one of the challenges endodontists face during root canal treatment and is of great importance due to its proximity to the gingival sulcus area and attachment loss, followed by bone loss [ 22 ]. The success of furcation perforation repair depends on the provision of a properly sealed crown restoration and the repair material’s resistance to displacement under masticatory forces and the condensing forces of the permanent restoration [ 23 ]. Amalgam condensation forces might reach 3.7–11.3 MPa, which is sufficient to remove the repair material from the furcation area [ 24 ]. Therefore, the bond strength of furcation repair material is an essential factor in clinical success. Various methods, such as tensile, compressive, and push-out bond strength tests have been used to investigate the bond strength. The push-out strength test is reliable, practical, and readily available [ 25 - 26 ].

MTA and CEM cement are among the materials that have exhibited successful outcomes in repairing furcation perforations since they are compatible with PDL and radiopaque, and have excellent stability, sealing ability, and marginal adaptation [ 7 , 27 ]. On the other hand, a change in the host tissue pH affects the physicochemical properties of these materials, leading to the loss of hardness and sealing ability, and a decrease in compressive strength [ 22 , 28 ]. In this study, to increase the efficiency of these materials, TiO_2_ nanoparticles were added. Because of the unique photoelectric properties and mechanical properties such as high elasticity, TiO_2_ nanoparticles are a suitable material to increase the efficiency and compressive strength of geometric materials [ 15 , 17 ]. This study aimed to evaluate the effect of incorporating TiO_2_ nanoparticles to white PC, white MTA, and CEM cement on the push-out bond strength in furcation perforation repair.

The results showed that, in general, all three substances exhibited the same bond strength in their pure form, with no significant difference between them, consistent with Tavasoli *et al*. [ 29 ] and lotfi *et al*. [ 30 ]. However, Sahebi *et al*. [ 31 ] demonstrated higher bond strength for CEM in comparison to MTA. On the other hand, Adl *et al*. [ 32 ] and Mohammadian *et al*. [ 33 ] reported higher bond strength for MTA in comparison to CEM as root end filling material. The noteworthy point is that, according to Ertas *et al*. [ 34 ] bond strength of MTA differs between various commercial brands. They showed that the push-out bond of the ProRoot MTA was statistically higher than MTA-Angelus. Furthermore, there was no significant difference between the bond strength of CEM cement and MTA-Angelus [ 34 ]. MTA -Angelus consists of 80% PC and 20% Bi2O3 and does not contain calcium sulfate, TiO_2_, P_2_O_5_, and FeO that are present in the composition of ProRoot MTA [ 9 , 34 ].

Furthermore, Regarding PC, Amoroso-Silva *et al*. [ 35 ] demonstrated that push-out bond strength of PC with 20% ZrO was similar to MTA. However, push-out bond strength of PC with 20% calcium tungstate was lower than MTA. It has been demonstrated that the retention of these materials to the dentinal walls depends on the water/powder ratio, temperature, humidity, the quantity of air trapped in the mixture, and the particle size of the materials, which might explain the different results obtained in different studies [ 5 ].

As an interesting finding in the present study, adding TiO_2_ to the MTA and PC increased the push-out bond strength. While, incorporation of TiO_2_ into CEM cement did not affect its push-out bond strength. Similarly, Bichile *et al*. [ 36 ] demonstrated that TiO_2_ added to MTA lead to a significant increase in its push-out bond strength. This increase in push-out bond strength in MTA is because of its unique pozzolanic activity property. Due to pozzolanic activity, the highly active TiO_2_ nanoparticles quickly consume Ca (OH)2. This reaction is favorable to form a denser structure of hydroxyapatite [ 36 ]. Hence, two events take place in this reaction. First, the amount of free Ca(OH)2 is eventually decreased. Second, when this reaction is taking place, there is an increase in the formation of calcium silicate hydrate and calcium aluminate hydrate. These hydration products are effective in increasing the overall strength of the material [ 36 - 38 ]. Furthermore, TiO_2_ nanoparticles would fill spaces between cement particles, producing smaller pores to increase the physical strength. Therefore, it is confirmed that the addition of nanoparticles to cement mortars improved their strength characteristics [ 37 - 38 ]. This is also in accordance with Samiei *et al*. [ 15 , 21 ] who concluded that addition of TiO_2_ nanoparticles in 1% weight ratio to MTA increases its push-out bond strength, compressive strength, working time and setting time.

Interestingly, Feng *et al*. [ 39 ] demonstrated similar results as observed for PC when combined with 1% weight TiO_2_ nanoparticles. They showed that achievement of good nanomodification with 1% weight TiO_2_ increased the amount of cementitious phase, decreased the microporosity and amount of internal microcracks and defects, and lead to the formation of a denser microstructure with reduced nanoroughness. They concluded that admixing TiO_2_ nanoparticles into PC not only lead to denser hardened cement paste but also altered the morphology and chemical compositions of cement hydration products [ 39 ].

On the other hand, according to the findings of the present study, the incorporation of TiO_2_ into MTA and PC gave rise to similar results, and CEM cement exhibited a different result, which might be attributed to the difference in the chemical composition of MTA and PC from CEM cement. According to previous reports [ 9 - 10 , 40 - 41 ], PC and MTA have the same composition and are composed of tricalcium silicate, dicalcium silicate, tricalcium aluminate, and bismuth oxide. However, CEM cement is chemically different from MTA. CEM is composed of calcium oxide, calcium phosphate, calcium carbonate, calcium silicate, calcium sulfate, hydroxide sulfate, and calcium chloride, and unlike MTA, it does not contain bismuth oxide. MTA contains calcium, silicon, and bismuth elements. However, CEM contains calcium, phosphorus, and sulfur elements. Although these two types of cement have different compositions, they have similar applications [ 9 - 10 , 40 - 41 ]. 

Considering the limitations of the present study, further studies are recommended to compare TiO_2_ nanoparticle modified CEM, MTA, and PC as a root-end filling material. Further studies are also recommended for long-term comparisons of these materials mixed with TiO_2_.

## Conclusion

The incorporation of TiO_2_ into MTA and PC increased their push-out bond strengths. However, adding TiO_2_ to the CEM did not affect its push-out bond strength.
